# Production Optimization of an Active *β*-Galactosidase of* Bifidobacterium animalis* in Heterologous Expression Systems

**DOI:** 10.1155/2019/8010635

**Published:** 2019-02-20

**Authors:** Xinxin Xu, Xiaohu Fan, Chao Fan, Xing Qin, Bo Liu, Chunming Nie, Ning Sun, Qingzhi Yao, Yuhong Zhang, Wei Zhang

**Affiliations:** ^1^Biotechnology Research Institute, Chinese Academy of Agricultural Sciences, Beijing 100081, China; ^2^College of Life Sciences, Inner Mongolia Agricultural University, Huhhot 010018, China

## Abstract

*β*-Galactosidase (E.C.3.2.1.23) catalyzes the hydrolysis of lactose into glucose and galactose and the synthesis of galacto-oligosaccharides as well. The *β*-galactosidases from bacteria, especially lactobacilli, and yeast have neutral pH and are much more likely to be developed as food additives. However, the challenges of cumbersome purification, product toxicity, and low yield in protein production have limited the commercialization of many excellent candidates. In this study, we identified a *β*-galactosidase gene (*bg42-106*) in* Bifidobacterium animalis* ACCC05790 and expressed the gene product in* Escherichia coli* BL21(DE3) and* Pichia pastoris* GS115, respectively. The recombinant bG42-106 purified from* E. coli* cells was found to be optimally active at pH 6.0 and 60°C and had excellent stability over a wide pH range (5.0–8.0) and at high temperature (60°C). The specific activity of bG42-106 reached up to 2351 U/mg under optimal conditions. The galacto-oligosaccharide yield was 24.45 g/L after incubation with bG42-106 at 60°C for 2 h. When recombinant bG42-106 was expressed in* Pichia pastoris* GS115, it was found in the culture medium but only at a concentration of 1.73 U/ml. To increase its production, three strategies were employed, including codon optimization, disulfide formation, and fusion with a Cherry tag, with Cherry-tag fusion being most effective. The culture medium of* P. pastoris *that expressed Cherry-tagged bG42-106 contained 24.4 U/mL of *β*-galactosidase activity, which is 14-fold greater than that produced by culture of* P. pastoris *harboring wild-type bG42-106.

## 1. Introduction

The *β*-galactosidase (EC 3.2.1.23) is an enzyme that breaks the *β*-1,4-d-galactosidic linkages of lactose to produce glucose and galactose. This enzymatic action makes it a food supplement to make lactose-free milk special for the lactose-intolerant people, which comprise more than 50% of the world's population [1]. Moreover, the enzyme has transglycosylation activity to synthesize galacto-oligosaccharides, the prebiotics to stimulate the growth and/or activity of beneficial bacteria in the colon [2]. Besides the application in food industry, *β*-galactosidase is attracting much attention owing to its ability to synthesize *β*-galactosyl derivatives that are involved in many biological processes [3]. Thus *β*-galactosidase represents a high-value biocatalyst, and its mass production is of importance for the commercial purpose.

Commercial *β*-galactosidases are mainly from microorganisms, especially from yeast and bacteria. Of them,* Kluyveromyces lactis* represents the major microbial source due to the high lactose hydrolysis activity of its *β*-galactosidase in milk. However,* K. lactisβ*-galactosidase has poor thermal and pH stabilities [4, 5] and is not a secreted protein, which means that its large-scale application is hampered by the high purification cost. Therefore, identification of *β*-galactosidases that have good stability and excellent catalytic properties is of practical significance. *β*-Galactosidases from many different species, including* Aspergillus niger* [6],* Thermus *sp. [7],* Lactobacillus reuteri* [8], and* Bifidobacteria infantis* [9], have been found. The excellent stability under a wide range of conditions and substantial catalytic activity make* Bifidobacteriaβ*-galactosidase a compelling candidate for application in food industry. The only bottleneck is its intracellular enzyme in nature, and its removal would present a further stage of cells disruption and purification, which are a costly process. Production of* Bifidobacteriaβ*-galactosidase in a recombinant expression system, e.g.,* Escherichia coli* or* Pichia pastoris*, may overcome these limitations.

For the study reported herein,* bg42-106* from* Bifidobacterium animalis* ACCC05790, which encodes a *β*-galactosidase with advantageous properties, was cloned and expressed in* E. coli *and* P. pastoris*. To improve gene expression and recombinant protein secretion in* P. pastoris*, several strategies were employed, including codon optimization and coexpression with a protein disulfide isomerase and fusion with a Cherry tag. Among these, codon optimization is widely and successfully used to increase the expression levels of foreign proteins in* P. pastoris *[10]. Protein disulfide isomerase is an endoplasmic reticulum-associated molecular chaperone involved in the rearrangement of incorrect disulfide pairings via its isomerase activity and also facilitates folding, assembly, and posttranslational modifications of eukaryotic proteins in general [11]. Several studies have shown that coexpression of* pdi* in* P. pastoris* improves heterologous protein expression [12, 13]. The commercial Cherry tag is a portion of the cytochrome heme-binding domain that can increase the solubility of the tagged protein. Our results showed that all these strategies increased the yield of* B. animalisβ*-galactosidase, with Cherry-tag fusion being most effective at protein secretion level.

## 2. Materials and Methods

### 2.1. Strains and Growth Conditions

*B. animalis* ACCC05790 from the Agricultural Culture Collection of China (Beijing, China) was grown anaerobically at 37°C in deMan, Rogosa and Sharpe (MRS) medium (2% glucose, 1% peptone, 1% meat extract, 0.5% yeast extract, 0.5% sodium acetate, 0.2% K_2_HPO_4_, 0.2% diammonium citrate, 0.1% (v/v) Tween 80, 0.02% MgSO_4_·7H_2_O, and 0.005% MnSO_4_·H_2_O, pH 6.2).* E. coli* strains TOP10 (TransGen, Beijing, China) and BL21(DE3) (Novagen, Darmstadt, Germany) were cultured in Luria-Bertani (LB) medium (0.5% yeast extract, 1% tryptone, and 1% NaCl) containing 100 *μ*g/mL ampicillin.* Saccharomyces cerevisiae* and* P. pastoris* GS115 were cultivated at 30°C in yeast extract/peptone dextrose medium (2% peptone, 2% glucose, and 1% yeast extract). Buffered glycerol complex medium, buffered methanol complex medium, regeneration dextrose medium, minimal dextrose medium, and minimal methanol medium were prepared according to manuals of* Pichia* expression kits (Invitrogen, Carlbad, CA).

### 2.2. Plasmids, Enzymes, and Chemicals

pEASY-T3 (Transgen) was used for gene cloning. pET-30a(+) (Novagen), pPICZA (Invitrogen), and pPIC9 (Invitrogen) served as expression vectors. Restriction and other enzymes used for DNA manipulations were obtained from TaKaRa (Dalian, China). Chemicals and reagents for high-performance liquid chromatography (HPLC) were purchased from Sigma (St. Louis, MO, USA) unless stated otherwise.* o*-Nitrophenyl-*β*-d-galactopyranoside (*o*NPG) was purchased from AppliChem (Gatersleben, Germany).

### 2.3. Cloning of the *β*-Galactosidase Gene* bg42-106*

Standard procedures for DNA extraction, plasmid isolation, restriction-enzyme digestion, and ligation were performed as described [26]. Bacterial *β*-galactosidase sequences were retrieved from GenBank by an Entrez search (http://www.ncbi.nlm.nih.gov/Entrez/); consensus regions in an alignment of these sequences were identified by ClustalW [27] and used to design the degenerate primers bG42F and bG42R (Table 1).

The core region of* bg42-106* was obtained via touchdown PCR with the* B. animalis* genomic DNA (50 ng) as the template and primers bG42F and bG42R (final concentrations, 5 *μ*M each primer). The PCR program was 94°C for 3 min; 10 cycles of 94°C for 30 s, 55°C for 30 s with a decrease of 1°C per cycle, and 72°C for 30 s; 30 cycles at 94°C for 30 s, 45°C for 30 s, and 72°C for 30 s. The gene fragment was purified using the reagents of a gel extraction kit (Tiangen, Beijing, China) and ligated into pEASY-T3 vectors, which were individually transformed into* E. coli* TOP10. Positive transformants were screened on LB agar plates containing 0.8 mg/mL X-gal, 3 mM isopropyl-*β*-d-thiogalactopyranoside (IPTG), and 100 *μ*g/mL ampicillin for 16 h at 37°C. White colonies containing the* bg42-106* fragment were confirmed by DNA sequencing. Based on the known sequence, the up- and down-stream flanking regions of* bg42-106* were obtained with genome-walking thermal asymmetric interlaced (TAIL)-PCR [28] with specific primers Dsp1, Dsp2, Dsp3, Usp1, Usp2, and Usp3 (Table 1).

### 2.4. Sequence Analysis

Verification of the nucleotide and deduced amino acid sequences, an open-reading frame search, multiple sequence alignment, and sequence assembly were performed using Vector NTI 10.3 software. The sequences of the DNA fragments obtained by touchdown PCR were compared with those of known *β*-galactosidases in GenBank by BLASTx. SignalP 3.0 (http://www.cbs.dtu.dk/services/SignalP) was used to determine if* bg42-106* contained a signal sequence.

### 2.5. Expression and Purification of Recombinant* bg42-106 *in* E. coli*

Primers bG42-106f and bG42-106r (Table 1) harboring the restriction sites* Eco*RV and* Not*I were used to amplify the full-length* bg42-106* from* B. animalis* genomic DNA. The PCR product was purified, enzyme digested, and inserted into pET-30a(+) to construct the recombinant plasmid pET30-*bG42-106*, which was further transformed into chemically competent* E. coli *BL21(DE3) cells by heat-shock at 42°C. Positive* E. coli *cells were cultured at 37°C in LB medium, 50 *μ*g/mL kanamycin until the OD_600 nm_ of the culture reached 0.6. The cells were then induced with 0.4 mM IPTG at 28°C for an additional 4 h. Recombinant bG42-106 carried a C-terminal (His)_6_ tag.

The cells were harvested by centrifugation (12,000 ×* g*, 5 min, 4°C), washed with 50 mM sodium phosphate (pH 6.5), and disrupted by ultrasonication. Cell debris was removed by centrifugation (12,000 ×* g*, 10 min, 4°C), and the crude enzyme extract was loaded onto a Ni^2+^-NTA affinity column (Qiagen, Hilden, Germany) that had been equilibrated with start buffer (20 mM sodium phosphate pH 6.5, 500 mM NaCl, and 5 mM imidazole). The protein was eluted at 1 mL/min with elution buffer (20 mM sodium phosphate pH 6.5, 500 mM NaCl, and 200 mM imidazole). Fractions containing *β*-galactosidase activity were pooled, desalted, and concentrated.

Protein concentration was determined using Bradford protein assay kit (Bio-Rad, Hercules, CA) with bovine serum albumin as a standard. The contents of total cellular protein and purified bG42-106 were examined by SDS-PAGE (12%, w/v). To estimate the native molecular mass, purified bG42-106 was detected in an 8% (w/v) nondenaturing PAGE. The activity was then examined by incubating the gel in an X-gal (4 mg/mL) solution.

### 2.6. *β*-Galactosidase Activity Assay

The *β*-galactosidase activity was monitored with* o*NPG hydrolysis at OD_420 nm_. Reactions contained 200 *μ*L of enzyme solution and 800 *μ*L of substrate (0.25% (w/v)* o*NPG in 100 mM Na_2_HPO_4_-citric acid, pH 6.0). Each reaction was incubated at 60°C for 15 min and terminated by addition of 1 mL of 10% (w/v) trichloroacetic acid and 2 mL of 1 M Na_2_CO_3_. One unit of *β*-galactosidase activity was defined as the amount of enzyme that liberated 1 *μ*mol of* o*-nitrophenol per min. The specific activity of the enzyme was expressed as units/mg protein.

### 2.7. Characterization of Recombinant bG42-106 from* E. coli*

To determine the pH optimum of bG42-106, its activity was measured at 60°C between pH 3.0 and 7.0 in 100 mM Na_2_HPO_4_-citric acid, pH 7.0 and 9.0 in 100 mM Tris-HCl, and pH 9.0 and 10.0 in 100 mM Na_2_CO_3_-NaHCO_3_. To measure its pH stability, the enzyme was first incubated in the aforementioned buffers at 37°C for 60 min and then assayed under standard conditions (pH 6.0, 60°C, 15 min). To determine the optimal temperature for enzyme activity, reactions were measured between 30 and 75°C at optimal pH for 15 min. The effect of temperature on stability was measured by incubating bG42-106 at 60°C or 65°C. Samples were removed at various times and assayed under standard conditions. To determine the half-life of bG42-106, the enzyme was incubated at 60°C, and activity was determined periodically for 72 h under standard conditions.

The effects of various reagents on enzyme activity were examined by assaying the enzyme in the presence of 1 or 10 mM of Na^+^, K^+^, Ca^2+^, Cu^2+^, Mn^2+^, Co^2+^, Cd^2+^, Fe^2+^, Ni^2+^, Mg^2+^, Zn^2+^, Pb^2+^, Ag^+^, Triton X-100, Na_2_EDTA, SDS, or CTAB in 100 mM Na_2_HPO_4_-citric acid, pH 6.0. Enzyme activity measured in the absence of any reagent was defined as 100%. Kinetic parameters (*K*_m_, *V*_max_, and *k*_cat_) were obtained from the Michaelis-Menten equation using GraphPad Prism software. The substrate (*o*NPG) concentration ranged from 1.38 to 25 mM. All experiments were carried out at 60°C and pH 6.0 in triplicate.

### 2.8. Lactose Hydrolysis and Galacto-Oligosaccharide Synthesis by Recombinant bG42-106 from* E. coli*

To assess the extent of lactose hydrolysis by bG42-106, reactions were performed at 50°C or 60°C in 100 mM Na_2_HPO_4_-citric acid (pH 6.0) containing 200 g/L lactose and purified bG42-106 for various times and terminated by heating for 10 min at 100°C. Formation of galacto-oligosaccharides and other saccharides was quantified by HPLC through a 6.5 × 300 mm Sugar-Pak I column (Waters, Milford, MA). This column can separate oligosaccharide molecules of the same size but different linkage type. Samples were eluted in 50 mg/mL CaNaEDTA at 500 *μ*L/min and 85°C. The yield of galacto-oligosaccharides (g/L) was calculated as [GOS] = [Lac_initial_] – [Lac_final_] – [Glu_final_] – [Gal_final_] [29], in which GOS is the yield of galacto-oligosaccharides, Lac_initial_ is the initial amount of lactose, Lac_final_ is the final amount of lactose, Glu_final_ is the final yield of glucose, and Gal_final_ is the final yield of galactose.

### 2.9. Expression of* bg42-106* in* P. pastoris*

Restriction enzyme-digested* bg42-106* as described above was ligated into pPIC9 to form the recombinant plasmid pPIC9-bG42-106, which was then transformed into* E. coli* TOP10 to maintain the plasmid. The construct pPIC9-*bG42-106* was linearized by* Bgl*II and then transformed into* P. pastoris* GS115 competent cells by electroporation. Transformed cells were selected according to the protocols in the* Pichia* expression kit manual (Invitrogen). Recombinant* P. pastoris *cells were incubated in 25 mL of buffered methanol complex medium at 30°C, followed by 48-h induction with 0.5% methanol. The culture supernatant was assayed for *β*-galactosidase activity, and the total protein in the culture medium was subjected to SDS-PAGE analysis. After disrupting cell integrity by grinding the cells in liquid nitrogen by metallic bead, intracellular *β*-galactosidase activity was also measured. The* P. pastors *strain containing the highest *β*-galactosidase was denoted as wild-type GS115/ bG42-106.

### 2.10. Codon Optimization and Expression of Codon-Optimized* bg42-106m*

To improve* bg42-106* expression in* P. pastoris*, low-usage (<15% frequency) codons were replaced with the common ones found in the* P. pastoris *genome [30]. In addition, the G+C content of* bg42-106* was adjusted to be similar to those of highly expressed* P. pastoris *genes. The optimized gene, denoted as* bg42-106m*, was synthesized by AugCt (Beijing, China).* bg42-106m *was ligated into pPIC9 and expressed in* P. pastoris* as described above. Intra- and extracellular *β*-galactosidase activities were assayed. The yeast strain containing* bg42-106m* was denoted as GS115/bG42-106m.

### 2.11. Coexpression of* scpdi* in* P. pastoris* GS115/bG42-106

The gene coding for the protein disulfide isomerase of* Saccharomyces cerevisiae* (*scpdi*) was PCR amplified with FastPfu DNA polymerase (Transgen) using* S. cerevisiae* genomic DNA as the template and primers ScPDIf and ScPDIr (Table 1). The PCR product was purified and ligated into pPICZA to form pPICZA-*scpdi*, which was transformed into* E. coli* TOP10 competent cells for sequencing. Recombinant pPICZA-*scpdi* was electroporated into* P. pastoris* GS115/bG42-106. The transformants were screened on yeast extract/peptone dextrose agar plates that contained 100 *μ*g/mL Zeocin. The* P. pastoris* strain that contained both* scpdi* and* bg42-106* was denoted as GS115/ScPDI-bG42-106. After coexpression of both proteins as described above for bGF42-106 expression, bG42-106 activity was measured as described above.

### 2.12. Fusion of* bg42-106* with a Cherry Tag

Primers CherryF and CherryR (Table 1) were used to clone the Cherry-tag coding sequence in the Cherry Express vector pSCherry1 (Delphi Genetics SA, Gosselies, Belgium). A 15-bp extension homologous to the sequence flanking the multiple cloning site in pPIC9-*bG42-106 *was added to both ends of the Cherry-tag sequence by PCR. The primer pairs P1 and P2 and P3 and P4 (Table 1) were used to individually amplify pPIC9/bG42-106. The 12- to 19-bp overlaps at the ends of the vector were used as the homologous recombinant arms. Using the CloneEZ recombinant cloning kit (Genscript, Nanjing, China), the Cherry tag and the vectors were fused via the Cherry-tag extensions and the homologous sequences in the vector to yield pPIC9-*Cherry*-*bG42-106*. In addition, a KEX2 site (Glu-Lys-Arg *∗* Glu-Ala-Glu-Ala, where *∗* is the cleavage site) coding sequence was inserted between* Cherry* and* bG42-106* genes for subsequent removal of the Cherry tag during secretion from* P. pastoris*. The plasmid pPIC9-*Cherry-bG42-106* was electroporated into* P. pastoris* GS115 as described above. *β*-Galactosidase activity in the culture medium and yeast cells was measured, respectively. The yeast strain containing pPIC9-*Cherry-bG42-106* is denoted as GS115/Cherry-bG42-106.

### 2.13. Nucleotide Sequence Accession Number

The nucleotide sequence for the* B. animalis* ACCC05790 *β*-galactosidase gene* bg42-106* was deposited in the GenBank database under accession number JX188444.

## 3. Results

### 3.1. Cloning and Sequence Analysis of* bg42-106*

The full-length* bg42-106* was obtained from the genomic DNA of* B. animalis* ACCC05790 through touchdown PCR and TAIL-PCR. The open-reading frame contained 2088 bp that encoded a polypeptide of 695 amino acids and a stop codon. No signal peptide sequence was identified. The calculated molecular mass of bG42-106 was 78 kDa. Sequence comparison indicated that bG42-106 belonged to family 42 of glycosyl hydrolases (GH). The deduced amino acid sequence was 98% and 33% identical to that of a putative *β*-galactosidase from* B. animalis* subsp*. lactis* HN019 (ZP_02963414) and a characterized *β*-galactosidase from* Bifidobacterium adolescentis *DSM20083 [16].

### 3.2. Expression and Purification of Recombinant bG42-106 from* E. coli*

**Figure 1:**
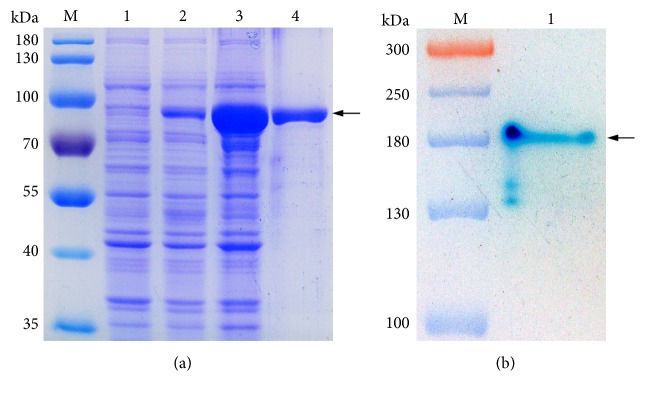
*Electrophoresis analysis of recombinant bG42-106 expressed in E. coli*. (a) SDS-PAGE profiles of recombinant bG42-106. Lanes: M, the protein molecular mass standards; 1, the lysate of induced* E. coli* BL2(DE3) harboring empty pET-30a(+); 2, the intracellular protein of uninduced* E. coli *BL21(DE3) harboring pET-*bg42-106*; 3, the intracellular protein from* E. coli* BL21(DE3) harboring pET-*bg42-106* with IPTG induction (4 mM and 28°C for 4 h); 4, the proteins eluted in 200 mM histidine wash from the Ni^2+^-NTA affinity resin. The arrow indicates the position of recombinant bG42-106. (b) Activity staining of purified bG42-106 on nondenaturing polyacrylamide gel. Lanes: M, the protein molecular mass standards; 1, purified bG42-106 from* E. coli*.

The recombinant plasmid, pET30-*bG42-106*, was constructed by cloning* bG42-106* into vector pET-30(+) and then transferred into* E. coli*. After induction with IPTG for 4 h, cells were sonicated, and the intracellular *β*-galactosidase activity was determined to be ~50 U/mL. The crude enzyme extract was purified to apparent homogeneity with Ni^2+^-NTA affinity chromatography (Figure 1(a)). Purified bG42-106 had an apparent molecular mass of ~80 kDa on SDS-PAGE, which was consistent with the theoretical mass of bG42-106 (78 kDa). The native molecular mass of bG42-106 was also analyzed, and the result showed that purified bG42-106 migrated one single band of ~180 kDa on native PAGE (Figure 1(b)), suggesting that bG42-106 existed as a homodimer in solution. It was higher than the theoretical value, which might be ascribed to the imprecision of the gel electrophoresis.

### 3.3. Characterization of Recombinant bG42-106 from* E. coli*

**Figure 2:**
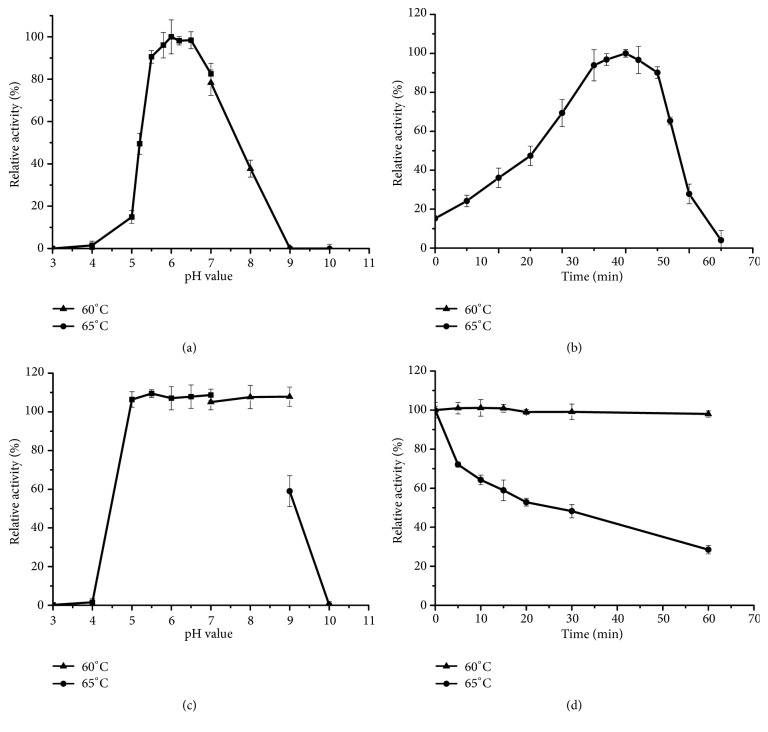
*Biochemical characterization of recombinant bG42-106 from E. coli.* (a) Effects of pH on the enzyme activity. (b) Effects of temperature on the enzyme activity. (c) pH stability of bG42-106. (d) Thermal stability of bG42-106. Closed squares, 100 mM Na_2_HPO_4_-citric acid (pH 3.0–7.0); closed triangles, 100 mM Tris-HCl (pH 7.0–9.0); closed circles, 100 M Na_2_CO_3_-NaHCO_3_ (pH 9.0–10.0). The error bars represent the mean ± standard deviation of three replicates.

The pH and temperature optima for bG42-106 activity were pH 6.0 and 60°C, respectively (Figures 2(a) and 2(b)). The enzyme retained almost all of the activity after incubation for 1 h between pH 5.0 and 8.0 at 37°C, but lost activity sharply below pH 4.0 or above pH 9.0 (Figure 2(c)). bG42-106 was thermostable at 60°C, retaining approximately 100% of the activity after 1-h incubation at 60°C and pH 6.0 (Figure 2(d)). The half-life at 60°C was determined to be 53.7 h. After incubation at 65°C for 30 min, the enzyme only retained about 50% activity.

The effects of various metal ions and reagents on bG42-106 activity were assessed (Table 2). Cu^2+^, Cd^2+^, Fe^2+^, and SDS (each at 10 mM) inhibited the enzymatic activity by 44% to 65%, but only SDS at lower concentration (1 mM) affected the activity. Conversely, 1 mM Pb^2+^ and Ag^+^ completely inhibited the activity. Other metal ions (K^+^, Na^+^, Co^2+^, Zn^2+^, and Ni^2+^) and reagents (CTAB and Na_2_EDTA) had no substantial effects on the activity.

With the use of* o*NPG as the substrate for a steady-state kinetic study, the *V*_max_, *K*_m_ and *k*_cat_/*K*_m_ values were found to be 720 *μ*mol/min/mg, 5.14 mM and 186.77 s^−1^ mM^−1^, respectively. The specific activity of purified bG42-106 was 2351 U/mg as determined under standard assay conditions.

### 3.4. Lactose Hydrolysis and Galacto-Oligosaccharide Synthesis of Recombinant bG42-106 from* E. coli*

**Figure 3:**
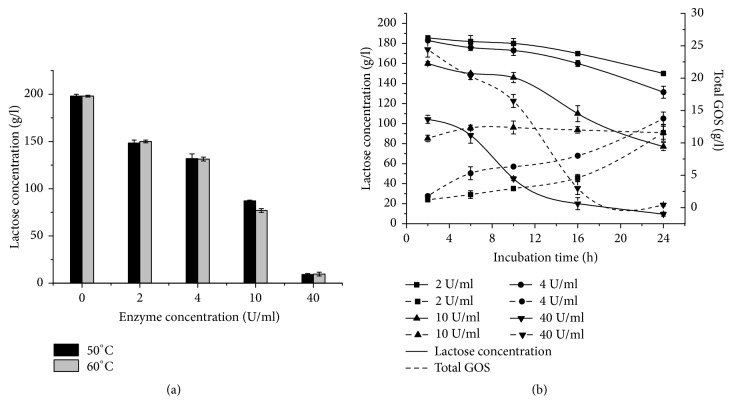
*The hydrolysis products of lactose (200 g/L) degraded by bG42-106*. (a) Effects of temperature and enzyme concentration on the lactose hydrolysis for 24 h. (b) Time course of lactose hydrolysis (solid lines) and galacto-oligosaccharide synthesis (dash lines) at 60°C with different enzyme concentrations. The error bars represent the mean ± standard deviation of three replicates.

The hydrolysis ability of bG42-106 to degrade lactose into galactose and glucose was assessed at two temperatures (50°C or 60°C) and different enzyme concentrations (2 to 40 U/mL) for 24 h. When the initial concentration of lactose was >200 g/L, hydrolysis was nearly complete after 24 h in the presence of 40 U/mL bG42-106 (Figure 3(a)). The rate of lactose hydrolysis was also monitored at different enzyme concentrations for up to 24 h (Figure 3(b)). The lactose concentration decreased from the initial 200 g/L to ~150 g/L at 24 h in the presence of 2 or 4 U/mL of bG42-106. At a concentration of 40 U/mL, however, bG42-106 degraded ~50% of the lactose within 2 h; then, the lactose hydrolysis rate decreased, and nearly ~90% of the lactose was hydrolyzed at 16 h.

The production of galacto-oligosaccharides in the presence of different amounts of bG42-106 was also assessed at 60°C (Figure 3(b)). In the presence of 2 or 4 U/ml of bG42-106 and 200 g/L lactose, the amount of galacto-oligosaccharides increased up to 15 g/L at 24 h. The maximal amount of galacto-oligosaccharides (24.45 g/L) was achieved with 40 U/mL bG42-106 at 60°C for 2 h. Thereafter, the amount of galacto-oligosaccharides decreased to almost zero at 24 h. When the bG42-106 concentration was 10 U/mL, a hydrolysis and synthesis balance of galacto-oligosaccharides was obtained, in which the amount of galacto-oligosaccharides was ~10 g/L.

### 3.5. Effect of Codon Optimization

**Figure 4:**
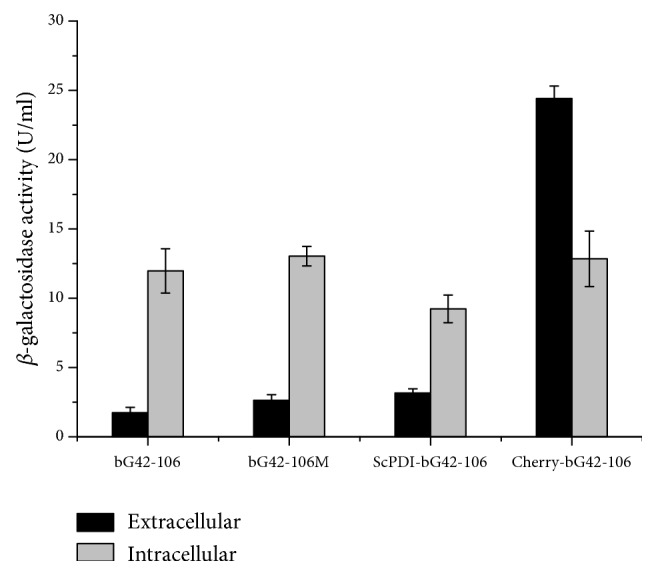
*The β-galactosidase activities of bG42-106 constructs in P. pastoris.* The error bars represent the mean ± standard deviation of three replicates.

A total of 451 nucleotides of* bg42-106 *involving 387 amino acids were replaced according to the optimized codon usage (Supplementary Table 1). The nucleotide sequence identity of wild-type and modified* bg42-106* was 77.9%. The G+C content was adjusted from 61.1% to 50.2% to mimic that of the* P. pastoris* genome*. bg42-106* and* bg42-106m *were expressed in* P. pastoris* GS115 with methanol induction. The highest enzyme activity found in the culture medium was 1.73 U/mL for bG42-106 and 2.64 U/ml for bG42-106m (Figure 4), respectively, reflecting a 1.5-fold improvement in the yield.

### 3.6. Effect of Coexpression of ScPDI and bG42-106

ScPDI and bG42-106 were successfully coexpressed in* P. pastoris*. After induction as described above, the *β*-galactosidase activity in the culture medium was 3.15 U/mL, 1.8 folds of that of bG42-106 alone. The intracellular *β*-galactosidase activity for ScPDI-bG42-106 was 9.23 U/ml, which was less than that of bG42-106 (11.97 U/mL) (Figure 4). ScPDI, therefore, acted as a molecular chaperone that may assist bG42-106 folding and subsequent secretion.

### 3.7. Effect of Fusing a Cherry Tag

A 326-bp Cherry-tag sequence was PCR amplified from pSCherry1 and fused with* bg42-106 *in vector pPIC9. The Cherry tag was removed from bG42-106 upon secretion. The extracellular activity of Cherry-bG42-106 was 24.4 U/mL, which was 14-fold greater than that for bG42-106. Moreover, the intracellular activity of Cherry-bG42-106 was less than that of its extracellular activity (Figure 4). The Cherry tag, therefore, significantly improved the yield of bG42-106 in* P. pastoris*. When bG42-106, bG42-106m, and bG42-106 were coexpressed with ScPDI, their intracellular levels were 3- to 7-fold higher than the extracellular ones (Figure 4).

## 4. Discussion

In this study we identified a *β*-galactosidase in* B. animalis* ACCC05790 and successfully produced it in both prokaryotic and eukaryotic expression systems. Compared with the *β*-galactosidases of various microbial sources that have been characterized (Table 3), bG42-106 under study represents relatively high *β*-galactosidase activity. Moreover, it has excellent enzyme properties, such as stability over a wide pH range (5.0–8.0) and at high temperature (60°C), and high yield of galacto-oligosaccharides (24.45 g/L). These features are particularly useful for biotechnological and industrial applications.

*P. pastoris* is well established for the rapid and cost-effective expression of recombinant proteins from discovery through commercialization. Proteins are secreted from* P. pastoris *under a strictly regulated control system. To improve the yield of bG42-106 in* P. pastoris*, we employed three strategies including codon optimization, coexpression of a protein disulfide isomerase, and fusion of a Cherry tag. Of them, protein disulfide isomerase has unpredictable effect on heterologous protein secretion from* P. pastoris *[31]. For example, coexpression of a protein disulfide isomerase increased the secretion of a malaria vaccine candidate (Pfs25) by 2- to 5-fold [32] and increased the yield of a human parathyroid hormone (hPTH) from 127 mg/L to 349 mg/L [33]. Because hPTH contains no cysteine, the protein disulfide isomerase may act as a molecular chaperone instead of a disulfide isomerase. In other studies, however, protein disulfide isomerase had little effect on the expression of targeted protein, and in one instance the production level of an immunoglobulin-binding protein was actually reduced [34]. bG42-106 contains 11 cysteines, some of which might form disulfides and thus require protein disulfide isomerase for formation of disulfide bonds, protein folding, and export from the cell. Thus we coexpressed bg42-106 and a protein disulfide isomerase from* S. cerevisiae* in* P. pastoris* and increased the yield of bG42-106 to 1.8 folds. Compared with previous studies, i.e., references [19, 20], the effect of protein disulfide isomerase on bG42-106 is less significant; it may be because this is not the most important limiting factor affecting the secretion of bG42-106 and other strategies are also expected to improve the yield of bG42-106.

Gene fusion, with glutathione S-transferase, maltose-binding protein, as examples, has been used to promote secretion of heterologous proteins in recombinant expression systems. Still, each fusion is assessed empirically, and thus the optimum results for any single case are not necessarily generalizable to other heterologously expressed proteins [35]. The Cherry tag, a red light-producing polypeptide composed of the cytochrome heme-binding domain, has been used to attain high levels of soluble protein in* E. coli* when fused to the N-terminus of a targeted protein. In some cases, Cherry tags have been used as a cost-efficient tool for protein purification by imparting heat stability [36]. In our study, the Cherry-tag gene was fused with* bg42-106* in vector pPIC9. Cherry-bG42-106 was secreted at a higher level, 14-fold greater than that produced by culture of* P. pastoris *harboring wild-type bG42-106. This favorable secretion level may be a consequence of the relatively high solubility of the Cherry tag itself.

In certain cases the conformation of an expressed protein may preclude its passage through cell membrane, suggesting the need to compare the conformation of the targeted protein with that of proteins secreted naturally. In addition, the expression of exogenous protein in* P. pastoris* usually depends on the natural host of the target gene; the wild-type unsecreted protein is often difficult to achieve secretion expression in recombinant host. bG42-106 from* B. animalis *ACCC05790 is an intracellular protein, which may explain its relatively low-level secretion in* P. pastoris* in the absence of the Cherry tag or another expression “trick” for heterologous proteins.

## 5. Conclusions

A novel *β*-galactosidase was identified in* B. animalis* ACCC05790 and expressed in both prokaryotic and eukaryotic systems. The recombinant protein showed high specific activity and good pH and thermal stability and produced high galacto-oligosaccharide yield. Three strategies including codon optimization, coexpression of a protein disulfide isomerase, and fusion of a Cherry-tag expression were carried out to promote the yield of bG42-106 from* P. pastoris*. Fusion protein approach improved the secretion level of Cherry-tagged bG42-106.

## Figures and Tables

**Table 1:** Primers used in this study.

Primers	Sequences (5′ 3′)_ _^a^
bG42F	GACTACWACCCNGANCANTG
bG42R	GTATTCRTTSTYNAYSTGCC
Dsp1	ACATCGTATCCCTGGCCATCTTCAG
Dsp2	ACCTCGCATCCGCCACCGCTTC
Dsp3	TCCGACATTCCGCACATACGCCC
Usp1	AGGGCGTATGTGCGGAATGTCGG
Usp2	AAGCGGTGGCGGATGCGAGGTC
Usp3	CTGAAGATGGCCAGGGATACGATG
bG42-106f	GCGC*CCTAGGGATATC*ATGTCAGCATCCACACAACATCGTG
bG42-106r	TATA*GCGGCCGC*GCGCCTGAACGCCAGAACGCCGTTTG
P1	CGTTTGGATCCTTCGAATAATTAGTTG
P2	CGGTCTCAGAAAAAGCATAAACAGTTCTAC
P3	AAAAGAGAGGCTGAAGCTATGAAAGCAAATATCAAATGGC
P4	TATGCTTTTTCTGAGACCGCAAAGTTGGTAGATGTGA
CherryF	CGAAGGATCCAAACGATGGCAGAACAAAGCGAC
CherryR	TCAGCCTCTCTTTTCTCAAGGGTTTCCGAAG
ScPDIf	AT*GAATTC*ATGAAGTTTTCTGCTGGTGCCGTC
ScPDIr	ATG*CGGCCG*CTTACAATTCATCGTGAATGG

^a^Y represents T or C, W for A or T, S for C or G, R for A or G, N for A, T, C, or G; restriction sites are italic; overlapping sequences are underlined.

**Table 2:** Effect of metal ions and chemical reagents on the *β*-galactosidase activity of purified recombinant bG42-106.

	Relative activity (%)_ _^a^	
Metal ions and reagents	1 mM	10 mM
Control	100.0	100.0
Na^+^	97.9 ± 1.0	95.6 ± 2.0
K^+^	98.6 ± 2.0	94.4 ± 1.0
Ca^2+^	97.6 ± 3.0	97.5 ± 1.0
Cu^2+^	90.6 ± 1.0	44.3 ± 4.0
Mn^2+^	100.9 ± 3.0	93.2 ± 2.0
Co^2+^	99.4 ± 2.0	96.0 ± 1.0
Cd^2+^	96.7 ± 2.0	59. 3 ± 2.0
Fe^2+^	96.9 ± 1.3	65.3 ± 3.0
Ni^2+^	102.3 ± 1.0	96.2 ± 0
Mg^2+^	102.3 ± 2.0	87.2 ± 1.0
Zn^2+^	104.2 ± 2.0	95.4 ± 2.0
Ag^+^	0	0
Pb^2+^	0	0
Triton	103.2 ± 4.0	94.1 ± 2.0
EDTA	100.3 ± 3.0	91.6 ± 2.0
SDS	55.5 ± 2.0	39.3 ± 4.0
CTAB	90.8 ± 1.0	91.8 ± 2.0

^a^Values represent the means of triplicates relative to the untreated control samples.

**Table 3:** Enzymatic properties of bG42-106 and its microbial counterparts.

Microbial source	pH_opt_	T_opt_ (°C)	Specific activity (U/mg)	References
*B. animalis *ACCC05790	6.0	60	2351	This study
*B. breve *B24	7.0	45	8073.60	[14]
*Thermus *sp. T2	6.5	80–90	900_ _^a^	[7]
*Paecilomyces aerugineus*	4.5	60	820	[15]
*B. infantis* HL96	7.5	60	569	[9]
*B. adolescentis *DSM20083	6.0	50	526	[16]
*Lactobacillus delbrueckii*	5.0–5.5	35–50	430	[17]
*Kluyveromyces fragilis*	6.5	—	250–290	[18]
*L. reuteri*	7.0	50	180–190	[8, 19]
*L. plantarum *WCFS1	7.5	55	154	[20]
*Bacillus stearothermophilus*	7.0	70	125	[21]
*Archaebacterium sulfolobus*	—	—	116	[22]
*Arthrobacter* sp.	7.0	18	115	[23]
*Halorubrum lacusprofundi*	6.5	50	110.83	[24]
*Aspergillus niger* van Tiegh	2.0–4.0	65	69.3	[6]
*Streptococcus mitis*	6.0–6.5	30–40	2.5_ _^a^	[25]

^a^ These data were converted from nmol for comparison purpose.

## Data Availability

No data were used to support this study.

## Supplementary Materials

Supplementary Materials
* Supplementary Table1*. Comparison of the codon usage of* bg42-106* and the optimized* bg42-106m* gene.
