# Supplementary material for: Production Optimization of an Active β-Galactosidase of Bifidobacterium animalis in Heterologous Expression Systems
Source: Biomed Res Int. 2019 Feb 20;2019:8010635. doi: 10.1155/2019/8010635 (PMC6402204; doi:10.1155/2019/8010635)
Supplement: Supplementary Materials — Supplementary Table1. Comparison of the codon usage of bg42-106 and the optimized bg42-106m gene. [file 8010635.f1.docx]

**Supplementary Table 1** Comparison of the codon usage of *bg42-106* and the optimized *bg42-106m* gene.

| Amino acid | Codon | RSCU^a^ | *bg42-106* | *bg42-106m* | Amino acid | Codon | RSCU | *bg42-106* | *bg42-106m* | Amino acid | Codon | RSCU | *bg42-106* | *bg42-106m* | Amino acid | Codon | RSCU | *bg42-106* | *bg42-106m* |
| --- | --- | --- | --- | --- | --- | --- | --- | --- | --- | --- | --- | --- | --- | --- | --- | --- | --- | --- | --- |
| Phe | TTT | 1.04 | 1 | 0 | Ser | TCT | 1.87 | 0 | 20 | Tyr | TAT | 0.89 | 3 | 0 | Cys | TGT | 1.30 | 1 | 3 |
|  | TTC | 0.96 | 31 | 32 |  | TCC | 1.34 | 13 | 20 |  | TAC | 1.11 | 17 | 20 |  | TGC | 0.70 | 10 | 8 |
| Leu | TTA | 0.94 | 0 | 0 |  | TCA | 1.06 | 4 | 0 | Stop | TAA | 1.36 | 0 | 0 | Stop | TGA | 0.55 | 1 | 0 |
|  | TTG | 2.10 | 3 | 43 |  | TCG | 0.45 | 9 | 0 | Stop | TAG | 1.09 | 0 | 1 | Trp | TGG | ∕ | 22 | 22 |
| Leu | CTT | 0.95 | 2 | 10 | Pro | CCT | 1.42 | 1 | 11 | His | CAT | 0.98 | 11 | 0 | Arg | CGT | 1.05 | 7 | 9 |
|  | CTC | 0.43 | 23 | 0 |  | CCC | 0.55 | 16 | 0 |  | CAC | 1.02 | 14 | 25 |  | CGC | 0.29 | 22 | 0 |
|  | CTA | 0.64 | 2 | 0 |  | CCA | 1.71 | 6 | 24 | Gln | CAA | 1.27 | 9 | 13 |  | CGA | 0.46 | 2 | 0 |
|  | CTG | 0.94 | 23 | 0 |  | CCG | 0.32 | 12 | 0 |  | CAG | 0.73 | 11 | 7 |  | CGG | 0.29 | 9 | 0 |
| Ile | ATT | 1.51 | 6 | 18 | Thr | ACT | 1.67 | 1 | 21 | Asn | AAT | 0.94 | 7 | 0 | Ser | AGT | 0.85 | 3 | 0 |
|  | ATC | 0.94 | 21 | 16 |  | ACC | 1.07 | 19 | 20 |  | AAC | 1.06 | 18 | 26 |  | AGC | 0.43 | 11 | 0 |
|  | ATA | 0.56 | 7 | 0 |  | ACA | 0.89 | 8 | 0 | Lys | AAA | 0.91 | 2 | 3 | Arg | AGA | 2.96 | 0 | 36 |
| Met | ATG | ∕ | 13 | 13 |  | ACG | 0.37 | 13 | 0 |  | AAG | 1.09 | 15 | 14 |  | AGG | 0.95 | 5 | 0 |
| Val | GTT | 1.81 | 5 | 24 | Ala | GCT | 1.93 | 3 | 50 | Asp | GAT | 1.10 | 16 | 15 | Gly | GGT | 1.88 | 5 | 30 |
|  | GTC | 0.95 | 12 | 21 |  | GCC | 0.98 | 36 | 15 |  | GAC | 0.90 | 44 | 45 |  | GGC | 0.52 | 30 | 5 |
|  | GTA | 0.54 | 5 | 0 |  | GCA | 0.93 | 18 | 4 | Glu | GAA | 1.17 | 18 | 17 |  | GGA | 1.30 | 7 | 10 |
|  | GTG | 0.70 | 23 | 0 |  | GCG | 0.17 | 12 | 0 |  | GAG | 0.83 | 25 | 26 |  | GGG | 0.30 | 3 | 0 |

^a^ RSCU (Relative synonymous codon usage) data was from Zhao et al. (2000)
